# Product Development Study of Freeze-Dried Apples Enriched with Sea Buckthorn Juice and Calcium Lactate

**DOI:** 10.3390/molecules30071504

**Published:** 2025-03-28

**Authors:** Marcellus Arnold, Wojciech Białas, Bartosz Kulczyński, Ribi Ramadanti Multisona, Joanna Suliburska, Michał Świeca, Aneta Wojdyło, Anna Gramza-Michałowska

**Affiliations:** 1 Department of Gastronomy Science and Functional Foods, Faculty of Food Science and Nutrition, Poznań University of Life Sciences, Wojska Polskiego 31, 60624 Poznań, Poland; marcellus.arnold@up.poznan.pl (M.A.); bartosz.kulczynski@up.poznan.pl (B.K.); ribi.multisona@up.poznan.pl (R.R.M.); 2 Department of Biotechnology and Food Microbiology, Poznań University of Life Sciences, 60637 Poznań, Poland; wojciech.bialas@up.poznan.pl; 3 Department of Human Nutrition and Dietetics, Faculty of Food Science and Nutrition, Poznań University of Life Sciences, Wojska Polskiego 31, 60624 Poznań, Poland; joanna.suliburska@up.poznan.pl; 4 Department of Biochemistry and Food Chemistry, University of Life Sciences, Skromna Str. 8, 20-704 Lublin, Poland; michal.swieca@up.lublin.pl; 5 Department of Fruit, Vegetable and Plant Nutraceutical Technology, Faculty of Biotechnology and Food Science, Wrocław University of Environmental and Life Sciences, 37 Chełmońskiego Street, 51-630 Wrocław, Poland; aneta.wojdylo@upwr.edu.pl

**Keywords:** apple, antioxidant activity, calcium, impregnation, osteoporosis, sea buckthorn

## Abstract

Enriched or fortified foods are typically linked to ultra-processed foods, limiting the choice of functional food in the market. Addressing the market potential, particularly the elder population with osteoporosis, the functional food industry should consider developing a healthy snack enriched with bioactive substances. This study aimed to produce freeze-dried Polish Gala apple with improved antioxidant properties and calcium content via impregnation or osmotic dehydration process. The solutions containing various concentrations of sea buckthorn (SB) juice and inulin were prepared at different temperatures and times, then analyzed by response surface regression modelling. Subsequently, the effect of the addition of 0–6% calcium lactate (CaL) on antioxidant properties and calcium content was also studied. Freeze-dried apple, after impregnation with 93.8% SB juice, 0:100 inulin–SB juice ratio, at 30 °C for 120 min, with the addition of 4% CaL (hereafter called “4% CaL” treatment), possessed a minimum yet acceptable loss of antioxidant properties and increased calcium content (2209.13 mg Ca/100 g). UPLC-PDA revealed the altered compositions of phenolics (flavonols were dominated by isorhamnetin-3-*O*-glucoside and isorhamnetin-3-*O*-rutinoside) and carotenoids in 4% CaL. The 4% CaL also exhibited lower polyphenol oxidase and peroxidase activities, moderate sensory acceptability with soft texture, and better nutritional values with lower calories when compared to the controls. This work is a scalable study, covering aspects of process design, physicochemical, nutritional, and enzymatic properties, as well as sensory profiling, which has potential for industrial implementation.

## 1. Introduction

In 2015, the United Nations declared 17 Sustainable Development Goals (SDGs), for which a target to reduce premature mortality from non-communicable diseases by one-third by 2030 through prevention and treatment and to promote mental health and well-being was mentioned in goal number three of the SDGs [1]. Functional food to prevent non-communicable diseases, particularly osteoporosis, has been developed recently. Among bioactive compounds, increasing intake of calcium and natural antioxidants, such as vitamin C, phenolics, and carotenoids, through functional food products is suggested to minimize the risk of osteoporosis, especially in the elderly [2,3].

Impregnation and osmotic dehydration (OD) processes have been studied over the past decade to improve the nutritional value (e.g., calcium and antioxidants) of various plant-based products, including, but not limited to, pumpkins, pineapples, apples, etc. [4,5,6]. During OD, mass transfer allows for the incorporation of solutes from a hypertonic solution to the food matrix, as well as for water removal from the food matrix as a drying pre-treatment. Although mass transfer in OD could help enzymatic browning inhibition and improve the flavor and taste of a product, the loss of the food matrix’s own solutes (i.e., vitamins, minerals, organic acids, etc.) may also happen during the process, which could be affected by the composition and concentration of osmotic agents, the physicochemical characteristics of the food matrix, and process parameters such as time, temperature, and agitation [7]. Various osmotic agents (i.e., salt, sugar, fruit juice concentrate) and process conditions of OD in fruits and vegetables have been reviewed previously [7].

Although the conventional OD process using sugars (sucrose and trehalose) promotes water loss, it significantly decreases the oxygen radical absorbance capacity (ORAC) value of dried strawberry and kiwi in comparison to untreated ones [8]. As opposed to sugar, inulin can be used as it has a lower caloric value than sugar and can act as prebiotic and dietary fiber [9]. The dried matter of strawberries after OD treatment using inulin only and inulin/chokeberry juice concentrate as osmotic solutions showed significantly higher polyphenol content and antioxidant activity via DPPH assay [10]. In OD-treated, oven-dried mango, using an emulsion prepared with inulin/piquin pepper oleoresin as an osmotic agent could recover the loss of free phenols, free flavonoid content, and antioxidant activity at certain levels, depending on temperature; as a comparison, using sucrose decreased their content and antioxidant activity significantly [9].

Recently, the utilization of various concentrated fruit juices, such as chokeberry, grape, apple, cranberry, etc., in OD treatment showed significant influence on the functional properties of various plant matrices [6,7,11]. Investigations of other fruit juices that are available in many regions and that are rich in bioactive compounds as potential osmotic agents are still needed. Sea buckthorn (SB) is a pro-health plant that is native to northwestern Europe through Central Asia to the Altai Mountains, western and northern China, and the northern Himalayas [12]. All parts of SB, especially its berries and juice, exhibit abundant bioactive compounds, including lipophilic (carotenoids and tocopherols) and hydrophilic antioxidants (flavonoids, tannins, ascorbic acid, phenolic acids), fiber, amino acids, and minerals (including calcium), offering a perspective on SB for upcoming research in the functional food field [13,14]. Thanks mainly to SB’s antioxidants, various pro-health benefits, such as anti-inflammatory activity, anti-hypertension properties, wound-healing activity, and protection against radiation, were observed [14]. SB fruit also possessed anti-osteoporosis effects as its active fractions promoted osteoblast differentiation in mesenchymal stem cells and increased osteogenic gene expression, which resulted in improved bone mineral density in an osteoporosis mouse model [15].

To ensure the effective intake of antioxidants and calcium by the population, potential food sources that are highly available in many regions should be taken into consideration; e.g., apples. Apples are one of the most popular fruits consumed worldwide thanks to their pleasant taste and high nutritional value; they are also one of the most cultivated crops in the world [16]. However, apples still lack calcium. Therefore, using SB juice and calcium as an osmotic or impregnation agent might be adapted to the apple to improve its calcium and antioxidant content in order to prevent osteoporosis. This study aimed to develop an alternative, locally sourced functional food product through the OD or impregnation process of apple flesh using SB juice, inulin, and calcium lactate. The suggested conditions to prepare the product were based on the antioxidant properties and calcium content of the final product. Some analyses, such as phenolic and carotenoid composition, sensory profiling, activity of polyphenol oxidase (PPO) and peroxidase (POD), and proximate analysis, were also conducted.

It is also worth noticing that the freeze-drying process was involved in this study. Freeze drying is an important and effective drying technique in the food industry, which has rapidly developed in the 21st century [17]. In comparison to other drying techniques, freeze drying provides significant advantages, such as preserving the quality of the food with minimum degradation of bioactive compounds or loss of nutritional value; easy reconstitution with a quick rehydration process; and lowering the moisture content, extending the shelf life of products. However, it also has some limitations, such as being time-consuming and costly. While Liu et al. [17] already reviewed some technical strategies (osmotic treatment, microwaving, etc.) to reduce the energy cost of the freeze-drying process with respect to various fruits and vegetables, the Food and Agriculture Organization suggested local sourcing as one of strategies to reduce costs in the food sector while creating a collaboration with local suppliers [18]. Our study is in agreement with this strategy, as utilizing local Polish apples and SB juice could be one of the ways to compromise the high production costs of freeze drying.

The outcome of this study could be beneficial for food producers, noting that consumer interest in functional food consumption is increasing. In contrast, enriched and/or fortified foods on the market are mostly associated with ultra-processed foods, such as fortified cereals, snack bars, etc., that contain a lot of additives and preservatives. This study may give new insight into the optimum conditions for producing freeze-dried apples enriched with bioactive compounds. This work is a scalable study, covering aspects of process design, physicochemical, nutritional, and enzymatic properties, as well as sensory profiling, which have potential for industrial implementation. Additionally, this research is also in line with the SDGs [1], especially goal number three, mentioned above, and number two supporting food security, as the product developed in this study was based on local and available resources—apple and SB juice. By applying responsible and sustainable production, it will be beneficial for the environment, such as minimizing transportation emissions, and additionally, it may strengthen the local economy by supporting regional farmers and producers.

## 2. Results and Discussion

### 2.1. First Part of Research

After the OD process using different process parameters and osmotic solution compositions (Table 1), total phenolic content (TPC) and antioxidant activities were analyzed by ABTS, DPPH, ORAC, and photochemiluminescence (PCL) of FA, FA + AA, and treated freeze-dried samples, and the results are displayed in Table S1. Generally, a higher concentration of SB juice resulted in higher TPC and antioxidant activities in treated freeze-dried samples, while increasing the inulin–SB juice ratio, regardless of the SB juice concentration and process parameters, resulted in lower TPC and antioxidant activities in freeze-dried samples. This might be because, during OD, the inulin was absorbed into the apples, which resulted in heavier freeze-dried products. Subsequently, lower concentrations of TPC and antioxidant activities per 100 g of freeze-dried products were obtained. Another possible mechanism is that hypertonic solutions, especially those with inulin, may force mass transfer, in which native phenolics leaked from the apples, while the SB juice from the solutions only partially recovered the loss of those apples’ native phenolics. This loss of antioxidant activity (ORAC) was also observed in OD of kiwi and strawberry using hypertonic sucrose or trehalose solutions [8]. In contrary, a study reported that the antioxidant activity by DPPH of frozen strawberry, osmotically dehydrated at 50 °C for 60 min, using 50 °Brix solutions of sucrose–inulin (1:1), inulin only, or inulin–chokeberry juice concentrate (1:4), increased by 26, 24, and 35%, respectively, in comparison to untreated strawberry, which was supported by increased TPC, expressed in gallic and chlorogenic acid equivalents in 100 g of dry matter [10]. This might be explained by the fact that apples and strawberries have different food matrices and the initial TPC of fruits may affect the final TPC after the OD process, noting that apples from different cultivars and regions exhibited different chemical compositions, including TPC, and antioxidant activities [16].

#### 2.1.1. Optimization of Conditions to Obtain Optimum Total Phenolic Content and Antioxidant Activities

Based on the results from Table S1, optimization of conditions to obtain the optimal TPC and antioxidant activities of treated freeze-dried apples was analyzed and is shown in Table 2. Inulin involvement was not suggested in terms of the optimized conditions, indicating that the inulin–SB juice ratio of 15:85 and 30:70 decreased the values of TPC and antioxidant activities (ABTS, DPPH, ORAC, PCL-ACL (lipid-soluble compounds) and PCL-ACW (water-soluble compounds)) in freeze-dried products, which is in accordance with the previous explanation (Section 2.1). This means that an inulin–SB juice ratio of 0:100 was the optimized one. Published studies on the effect of inulin as an osmotic agent in osmotically dehydrated apples have been limited to the mass transfer phenomenon [19] and reduction in sugar uptake [20], rather than antioxidant activity or retention of the contents of bioactive compounds. However, in contrast, for other matrices, like freeze-dried strawberries after OD with a chokeberry juice concentrate–inulin (1:4 *w/w*) solution, the results exhibited about three times higher TPC and about two times higher DPPH antiradical activity than in freeze-dried control samples [21]. Additionally, the authors also observed similar TPC and DPPH antiradical activity in freeze-dried strawberries treated with 50% sucrose, 50% inulin, and a 50% sucrose–inulin mixture (1:1, *w*/*w*) solution. OD of mango using inulin and piquin pepper oleoresin also resulted in higher free phenol, flavonoid, and DPPH radical scavenging activity than when using a sucrose solution, especially at 40 °C; however, the free phenols and flavonoids were lower in comparison to the untreated control [9].

Furthermore, water, ranging from 1.2 to 31.9% (3.08 to 79.65 g in 250 g), was, interestingly, suggested in the composition of osmotic solutions in all TPC and antioxidant activities, except PCL-ACW (0% or 0 g in 250 g) (Table 2). In other words, SB juice at a concentration of 68.1–100% in water was suggested, depending on the type of assay. The addition of water may affect the total soluble solid (°Brix, Table 1) of the solution, which then affects mass transfer during the OD or impregnation process. The higher Brix value in osmotic solutions than in the food matrix not only improves the uptake of solutes from osmotic solutions, but also lets the water and solutes from the food matrix pass into the osmotic solution [22]. This may indicate that a higher total soluble solid or concentration of SB juice in the osmotic solution does not always improve the TPC and antioxidant activities of treated freeze-dried apples, as the loss of apples’ solute or phenolics could also occur (Section 2.3.1). Similar results were reported by Kowalska et al. [23], as convective-dried Idared apples, osmotically dehydrated by 25 °Brix concentrated apple juice (CAJ), showed higher TPC than those treated with 50 °Brix CAJ, irrespective of the temperatures applied (40 and 60 °C); and those treated with 25 °Brix CAJ resulted in a 9–24% decrease in comparison to untreated dried apples. However, when concentrated cherry juice (CCJ) only, or in combination with fructooligosaccharide (CCJ-F) at 25 and 50 °Brix, was used, the TPC of treated dried apples increased with an increasing of the Brix value of CCJ or CCJ-F [23]. Therefore, the fruit juice type could affect TPC and, thus, the antioxidant activities of treated dried apples.

Regarding treatment time (Table 2), the optimized time was about 120 min for ABTS, DPPH, ORAC, PCL-ACW, and PCL-ACL, while for TPC, 73.54 min was the optimized time. The trend in TPC is consistent with previous work, as the dried Champion apple showed increased TPC during 90 min of OD using concentrated chokeberry juice, and then decreased about 8% at 120 min [24]. Regarding temperature (Table 2), the optimized temperature was about 30 °C for ABTS, DPPH, ORAC, PCL-ACW, and PCL-ACL, while 38.82 °C was the optimized temperature for TPC. A higher temperature might either positively or negatively affect mass transfer during the OD or impregnation process [25]. Higher temperatures may accelerate water loss and solid gain during OD, which then may increase the nutritional value of the end products; however, a higher temperature may also decrease the antioxidant activity as degradation of phenolics may occur, as well as leaching of products, in this case the apple’s phenolics. A higher temperature can also reduce the viscosity of the osmotic solution and thus affect mass transfer [25]. Similar results were also found with respect to oven-dried mango slices, osmotically dehydrated using an emulsion of inulin and piquin pepper oleoresin, where treatment at 40 °C showed higher TPC and antioxidant activity than treatment at 30 and 50 °C [9].

The predicted and validation mean values of the optimized conditions in each assay were evaluated (Table 2). The validation mean values of all antioxidant activities (ABTS, DPPH, ORAC, PCL-ACL, and PCL-ACW) fall within the range of 95% prediction and confidence intervals, indicating that the predicted optimized conditions are consistent with the validation of observed data. In contrast, TPC’s validation mean value falls outside both intervals, which might be caused by the limitations of the Folin–Ciocalteu assay. It is a simple, reproducible, robust, and comparable assay, yet it is sensitive to pH, temperature, and reaction time. This assay also includes non-phenolic reducing agents, such as reducing sugars, some amino acids, organic acids, ascorbic acid, etc., that could interfere with the TPC results [26,27]. Nevertheless, TPC still showed high and significant positive correlations (*p* < 0.05) with all antioxidant activities, for which Pearson’s coefficients ranged from 0.91 to 0.98 (Table S2).

Among the optimized conditions, the conditions to obtain the optimum ORAC value (Opt_ORAC; 93.8% SB juice in water, 0:100 inulin–SB juice ratio, 30 °C, 120 min, based on Table 2) were chosen for the second part of the research, as the ORAC assay uses peroxyl radicals, which are biological relevant free radicals [26]. Freeze-dried apples prepared in Opt_ORAC conditions showed significantly higher ORAC values (3532.58 mg TE/100 g product) than FA (3423.52 mg TE/100 g product, *p* < 0.05), yet still similar to FA + AA (3579.68 mg TE/100 g product, *p* > 0.05) (Table 2). This means that freeze-dried apples prepared in Opt_ORAC conditions involving SB juice could recover the loss of antioxidant activity during the treatment, especially when compared to treatments with 0–50% SB juice and inulin–SB juice ratios of 15:85 and 30:70 at different temperatures and times (Table 1 and Table S1).

#### 2.1.2. Color of Freeze-Dried Apples Prepared in Optimized Conditions

Visual perception, such as perception of color, plays an important role in the selection of healthy food products by consumers. The appearance, L, a, and b values, and ΔE, BI, WI, and YI of freeze-dried apples prepared in optimized conditions (Opt_TPC, Opt_ABTS, Opt_DPPH, Opt_ORAC, Opt_PCL-ACL, and Opt_PCL-ACW) and of the controls (FA and FA + AA) were measured and described in Table 3. FA + AA possessed the highest L value (81.06), higher than FA (70.96) and optimized samples (45.14–62.19). Dipping apples in an ascorbic acid solution prevents the melanin formation of apples by binding to intermediate products, e.g., *o-*quinone, which results in a higher lightness [28]. Furthermore, compared to the controls (FA and FA + AA), all optimized freeze-dried samples showed lower WI (ranged from 24.61–33.58) and higher a values (redness; 20.18–23.79), b values (yellowness; 45.71–50.74), ΔE (35.20–45.43), BI (165.13–248.64), and YI (116.57–145.74). However, the higher ΔE, BI, and YI are not always related to higher enzymatic browning activity as it can be caused by the color of dipping solutions [29]. In this case, the phenomenon was caused by the orange color of SB juice in this study (L value: 41.77 ± 0.19; a value: 23.34 ± 0.03; b value: 47.92±0.43), noting that SB juice contains carotenoids [14]. Polyphenol oxidase and peroxidase activities were observed to confirm the effect of SB juice impregnation on the enzymatic browning activity of treated freeze-dried apples (Section 2.3.3.). The orange color of the treated product could also be an alternative in terms of producing a functional food with appealing color, particularly for kids.

Different types of fruit juice (apple, grape, and cranberry) used in the OD process also affected the color of hot-air-dried apple chips [6]. However, the authors performed the hot-air drying process at 70 °C, which may have also influenced the final color of the apple chips through enzymatic browning, Maillard reaction, and ascorbic acid oxidation. In the present study, a non-thermal freeze-drying process was performed to prepare the end products, thus those thermal-related factors may not have influenced the color of treated freeze-dried apples. 

### 2.2. Second Part of Research

**Figure 1:**
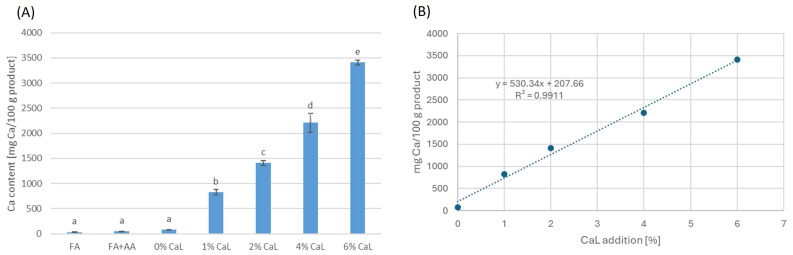
The effect of calcium lactate addition on the calcium content of freeze-dried controls (FA and FA + AA) and treated freeze-dried apples: (**A**) calcium content; (**B**) linear regression analysis (0–6% CaL addition). Different lowercase letters in (**A**) indicate significant differences (*p* < 0.05) between samples.

In the second part, the conditions of Opt_ORAC from the first part were applied, with the addition of 0–6% CaL. The effect of CaL addition on the TPC, antioxidant activities (Table 4), and calcium content (Figure 1) of treated freeze-dried apples was observed. In Table 4, increasing the CaL concentration resulted in significant decreasing values of TPC and all antioxidant activities in treated freeze-dried apples. The highest decrease was found in freeze-dried apples prepared with 6% CaL, which possessed 38, 37, 24, 45, 35, 56, and 45% lower values of TPC, ABTS, DPPH, ORAC, PCL-ACL, PCL-ACW, and PCL-IAC, respectively, than those prepared with 0% CaL. This phenomenon may be caused by interaction between bioactive compounds, such as phenolics and non-reactive redox metal–calcium. A previous study showed that in food and beverages, calcium may the lower antioxidant activity of plant phenolics through complexation or by precipitation [30]. Additionally, the decrease may also be explained in terms similar to the case of inulin in Section 2.1, in which CaL was adsorbed in the apple flesh during the impregnation process, and subsequently, heavier freeze-dried products were obtained and lower TPC and antioxidant activities were observed per 100 g of end products. The increased weights of freeze-dried samples: 10.9, 19.9, 31.4, and 37.0% were observed in samples at 1, 2, 4, and 6% CaL, respectively, in comparison to the weight of 0% CaL sample.

Those results were also supported by the phenomenon in which the addition of 1–6% CaL significantly increased the calcium content of the freeze-dried apples, accounting for 828.11–3408.04 mg Ca/100 g of product (Figure 1A). The impregnation process promoted the adsorption of CaL, and subsequently, the calcium content increased in the freeze-dried products. In other studies, an increase in calcium content was also found in OD-treated plant matrices using a solution with CaL. Tappi et al. [31] observed increased calcium content in OD-treated apples in solutions containing 40% sucrose and 4% CaL, with and without 2% ascorbic acid. For OD of more than 2 h, samples treated in a solution with ascorbic acid showed a significantly higher increase in calcium content than those treated in a solution without ascorbic acid. In a study of pineapples, 6 h of OD using solutions of (a) 40% sucrose with 4% CaL and (b) 50% sucrose with 4% CaL resulted in pineapples with approximately 90 mg Ca/100 g of wet basis [4].

Furthermore, it was also noticed that at 0% CaL (Figure 1A), the calcium content was 79.44 mg Ca/100 g of product, which is about 2.4 and 1.8 times higher than that of FA and FA + AA, respectively, yet they were similar (*p* > 0.05). Despite the insignificance, this increase might be explained in terms of the SB juice used in the osmotic solution of this study, which also contains 12.7 mg Ca/100 g. The calcium content of the SB juice was slightly higher but comparable to the total in a review by Ciesarová et al. [13], who observed that the calcium content of SB juice ranged from 2.1 to 10.9 mg Ca/100 g, depending on the country of origin. Additionally, from the calcium content of samples prepared at 0–6% CaL, a linear regression model (Figure 1B) was obtained with the equation of [Ca content in mg Ca/100 g] = 530.34 [%CaL addition] + 207.66 (R^2^ = 0.9911).

Besides antioxidant properties, the calcium content of end products was also considered. It was observed that the TPC and antioxidant activities, except PCL-ACW, of the samples prepared with 2 and 4% CaL were similar (*p* > 0.05) (Table 4). Noting that the freeze-dried apples prepared at 4% CaL contain 2209.13 mg Ca/100 g, which was 1.57 times higher than those prepared at 2% CaL, the suggested CaL concentration is 4% CaL. The current daily recommended dietary allowance (RDA) of calcium is 1000 mg Ca for adults 19–50 years of age; absorption of dietary calcium in adults is 25%, which decreases with age [32]. By assuming an absorption of 25%, consuming 100 g of freeze-dried apples prepared after impregnation using 93.8% SB juice in water, at a 0:100 inulin–SB juice ratio, with the addition of 4% CaL at 30 °C for 120 min (hereafter called 4% CaL treatment), could fulfill 55.2% of the RDA, with a minimum loss of antioxidant activity and TPC in the end products. The total soluble solid of this suggested solution was 8.63 ± 0.06 °Brix, which was slightly hypotonic when compared to FA + AA (10.13 °Brix) during the treatment process. Therefore, the term impregnation is used hereafter instead of OD, as with OD, mass transfer takes place when water from the food matrix passes into the hypertonic solution through apoplastic, symplastic, and transmembrane transports [7].

### 2.3. Analyses of Selected Samples

Based on the results of the first and second parts of the research, the freeze-dried products following 4% CaL treatment and the controls (FA and FA + AA) were selected for further analyses.

#### 2.3.1. Quantification of Phenolic and Carotenoid Composition of Freeze-Dried Apples by UPLC-PDA

The quantification of phenolic and carotenoid content in selected freeze-dried samples (FA, FA + AA, and 4% CaL) by the UPLC-PDA method is shown in Table 5. Phenolic substances aid in the prevention and treatment of osteoporosis not only by inhibiting oxidative stress, but also by affecting bone metabolism, reducing bone resolution, maintaining bone density, and lowering osteoclast differentiation [33]. In terms of phenolics, flavan-3-ols dominated the phenolic composition of FA and FA + AA at 51.7 and 54.8%, respectively, followed by phenolic acids, which accounted for about 38% in each sample, while flavonols (46.36 mg/100 g product) dominated the phenolic composition of the 4% CaL sample at 43.5%, followed by flavan-3-ols at 41.7%. The 4% CaL sample had a significantly higher content and more types of flavonols than FA (1.07 mg/100 g product) and FA + AA (0.96 mg/100 g product). Among flavonols, only quercetin-3-*O*-rhamnoside (compound 21) was found in all samples, while the rest were found only in 4% CaL. Impregnation using SB juice in the 4% CaL sample enriched the flavonols of the final product. The flavonol composition of 4% CaL was in agreement with that of the berries of six Polish SB cultivars [34], where compounds 10–24 were also found, with isorhamnetin-3-*O*-glucoside and isorhamnetin-3-*O*-rutinoside as the two dominant compounds. Those two compounds were also considered as major flavonol glycosides in SB berries from Canada, Finland, and China [35]. Flavonol glycosides in SB have been studied with respect to prevention of chronic diseases, including diabetes, cancer, and cardiovascular disease [36]. Despite the increase in flavonol content, 4% CaL had 13.96 mg of phenolic acids, which was about 60–64% lower than FA and FA + AA (*p* < 0.05). The sum of dihydrochalcones in 4% CaL was also significantly lower than the sum of dihydrochalcones in the control groups, while for flavan-3-ols, 4% CaL possessed a higher catechin and procyanidin B1 content yet lower epicatechin and procyanidin C1 compared to the controls. The highest total phenolic content was found in 4% CaL (106.49 mg/100 g product), about 3.3% and 19.2% higher than FA + AA and FA, respectively. The total phenolic trend via the UPLC-PDA method was generally different for TPC and antioxidant activities via the Folin–Ciocalteu method, where the TPC and antioxidant activities of 4% CaL were lower than the controls, yet it is worth noticing that the UPLC-PDA and Folin–Ciocalteu methods are two different methods with their own advantages and limitations. Interference from non-phenolic compounds may overestimate TPC in the Folin–Ciocalteu method [26]; in this case, most likely from ascorbic acid. Liquid chromatography focuses only on specific or determined compounds of interest, and there might be other compounds that are not covered [37] in this study; for example, non-glycoside flavonoids (aglycones). It was also found that among all apple cultivars, the flesh of Oldenburger had the highest TPC and antioxidant activities (ABTS and ORAC), yet total polyphenols via high-performance liquid chromatography with diode array detection were not the highest [38]. Another limitation of the Folin–Ciocalteu method is also gallic acid usage as a standard, which reacts more strongly than other phenolics and thus may overestimate TPC [37]. The principle means of quantifying TPC in the Folin–Ciocalteu and liquid chromatography methods are also different; the first one focuses on reduction reactions using tungsten and molybdenum-containing reagents and is spectrophotometrically evaluated, while the second one focuses on separation and quantification of analytes present in the samples [39]. Additionally, another study observed weak correlations between the sum of flavonols and antioxidant activities by ABTS, DPPH, and FRAP methods in various apple cultivars in Poland, whereas the sum of flavan-3-ols and the sum of hydroxycinnamic acids exhibited higher positive correlations with antioxidant activities [40].

Carotenoids have been shown to have antiosteoporotic effects and a bone-building support effect [3]. The 4% CaL sample possessed a total of 38.63 mg of carotenoids per 100 g of product, about 17 times higher than that of the controls, which could be caused by the involvement of SB juice during the impregnation process. Carotene dominated the carotenoids in 4% CaL at 74%. It is worth noting that the composition of carotenoids varied with respect to commercialized SB juice in Poland [41]. Furthermore, the totals and compositions of carotenoids in FA and FA + AA in this study were comparable to the results observed previously, particularly for Royal Gala flesh [42,43].

In this study, phenolic and carotenoid compositional changes in freeze-dried apples were affected by both the impregnation process using SB juice and CaL and also by dipping in an ascorbic acid solution, which then may influence the properties of end products.

#### 2.3.2. Sensory Profiling of Freeze-Dried Apples

**Figure 2:**
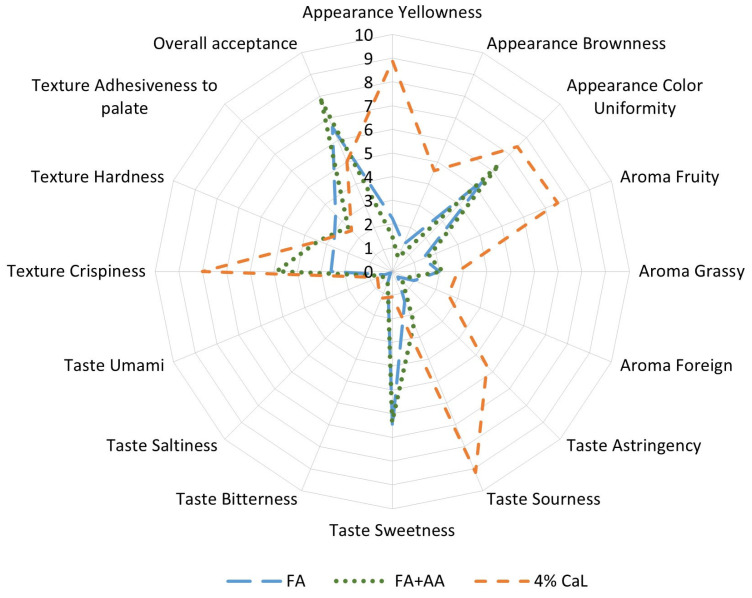
Sensory profiling of freeze-dried apples: FA, FA + AA, and apples treated using Opt_ORAC conditions (93.8% SB juice in water, 0:100 inulin–SB juice ratio, 30 °C, 120 min) with the addition of 4% CaL. Scores 0–10 for descriptors of each attribute are described in Table S3.

In the development of functional food products, it is necessary to ensure that the products are not only nutritionally beneficial and promote health but also sensorially acceptable to consumers. Therefore, three selected freeze-dried samples (FA, FA + AA, and 4% CaL) were evaluated via sensory profiling and overall acceptance (Figure 2). For the attribute of appearance, 4% CaL was profiled as having darker yellowness, brownness, and higher color uniformity in comparison to FA and FA + AA as controls, noting that 4% CaL used SB juice containing carotenoids during its preparation. It is also worth noticing that the stability of carotenoids is influenced by environmental factors, including pH. A study reviewed that depending on the food matrix, acidic pH may either improve (due to the higher solubility of carotenoids) or decrease (due to isomerization) the intensity of the yellow-orange color of a food product [44]. Furthermore, the lightest brownness and yellowness were found in FA + AA, which was influenced by ascorbic acid and enzymatic browning inhibition. The utilization of SB juice might also be responsible for the more intense grassy and sea buckthorn aroma in 4% CaL compared to the controls. Despite the foreign aroma being more noticeable at 4% CaL, the score was rather low (2.55). In terms of taste, the 4% CaL samples possessed a high sourness intensity, a rather high astringency intensity, and a low bitterness and sweetness intensity. This was in accordance with a previous study, as sensations of astringency and sourness were more noticeable than sensations of sweetness and bitterness in SB puree from various cultivars [45]. Malic acid contributes to the sourness, while quinic acid is responsible for the astringency of SB berries [14]. Additionally, tastes of umami and saltiness were negligible, as the scores were below 1 in all samples. For texture, the addition of CaL in the 4% CaL treatment might play a significant role in terms of improving its crispiness (8.00), which was higher in comparison to the controls. CaL could form complexes with carboxyl groups of pectin, which is available in apples, and thus affect the firmness and texture of the product [25]. Furthermore, 4% CaL was also as soft as the controls and showed low adhesiveness to palate, which is then suitable for the elderly, who are prone to osteoporosis, noting that foods with soft texture and low adhesiveness could be easily swallowed by them [46]. For overall acceptance, 4% CaL was moderately acceptable (5.02) to the panelists, but still showed a lower score than FA (6.60) and FA + AA (7.82). The lower overall acceptability of 4% CaL might be caused by the imbalanced taste of SB juice as one of the ingredients, especially the high sourness intensity. This was supported by a previous study in which most of the panelists described the apple chips as crispy and sweet with a sourish apple taste [47]. Therefore, improvements in sensory properties should be investigated in further studies involving inulin, for example, but with an inulin–SB juice ratio lower than 15:85, as inulin could also increase the sweetness but with lower calories than sugar. Furthermore, the means of consuming this 4% CaL sample could also be modified—for example, as a topping for yogurt, ice cream, or fruit salad—in order to perceive a more balanced flavor, instead of it being consumed directly as chips. If necessary, involving safe and natural fruit flavors in the formulation may also compromise the sourness and astringency of the product, achieving more balanced sensory properties. Further studies on sensorial improvement without significantly deteriorating the health benefits of the product are encouraged.

#### 2.3.3. Activity of Enzymes

The PPO and POD activities of FA, FA + AA, and 4% CaL were also measured (Table 6). The FA + AA and 4% CaL significantly reduced 13.7 and 88.9% of PPO activity in comparison to FA. For POD activity, the 4% CaL treatment inhibited 43.2% of POD activity in comparison to FA. In the preliminary study, it is worth noting that the SB juice used in this research did not show either PPO or POD activity, which is as expected as the SB juice was pasteurized and might denature the enzymes. Therefore, the inhibition of PPO and POD activities in 4% CaL might be caused by the presence of SB juice, which, based on previous studies, contains lipophilic (carotenoids and tocopherols) and hydrophilic antioxidants (flavonoids, tannins, ascorbic acid, phenolics) [13,14] and may inhibit PPO and POD activity in apple products [29]. It is also worth noting that in Table 5, the sum of phenolic acids in FA and FA + AA were higher than in 4% CaL, which then may act as substrates of PPO and POD and could stimulate enzymatic browning. However, the relationship between phenolic acids and prevention of enzymatic browning is also cultivar-specific; some cultivars may have high phenolic acid content but low PPO and POD activities, while others may show the opposite effect [29].

The POD activity of FA + AA was more than twice as high as that of FA. A similar result was found in a previous study [48], where dipping Fuji apple slices in 1% ascorbic acid for 1 min increased the POD activity of the slices, while for solutions that combined ascorbic acid with other anti-browning agents, inhibition of POD activity was observed. The authors explained that ascorbic acid may also act as a pro-oxidant and subsequently exhibit the opposite effect with respect to hydrogen peroxide and peroxidation.

The results observed in this study provided information with respect to the impregnation of apple flesh using SB juice and calcium lactate, which inhibited the PPO and POD activity of the resulting freeze-dried apples. However, the mechanism of inhibition and application in terms of other SB cultivar juices should be further studied. To date, according to our knowledge, the utilization of SB juice to prevent PPO and POD activity in plant matrices has not been carried out. Tkacz et al. [49] investigated other enzymatic activity of Polish cultivars with respect to SB fruit extracts, such as anti-α-amylase, anti-glucosidase, anti-lipase, and anti-lipoxygenase activity, which indicated that SB fruit may potentially be of benefit to general health. Instead of SB juice, in the preliminary study of Zhang et al. [50], the authors found that SB fruit extract showed an anti-browning effect in fresh-cut potato sticks based on ΔE and sensory analysis in comparison to the control group (water-treated). However, the effect was still weaker than for SB leaf extract [50]. The SB leaf extract was also incorporated into a carboxymethyl cellulose-based edible coating, which successfully inhibited the browning index, PPO, and POD activities in apples during storage [51]. Therefore, the potency of SB juice as a natural anti-browning agent, especially in terms of its mechanism of inhibition, could be further investigated in the future.

#### 2.3.4. Proximate Analysis of Freeze-Dried Apples

The proximate analysis of FA, FA + AA, and 4% CaL is shown in Table 6. In comparison to the controls, 4% CaL freeze-dried apples exhibited significantly higher protein and lipid content, with lower carbohydrate content (*p* < 0.05). This could be due to the involvement of SB juice in the 4% CaL samples during the treatment. Dong et al. [14] reported that SB berry contains 3% lipids and major fatty acids, such as palmitoleic (32–52%), palmitic (26–36%), oleic (10–26%), linoleic (5–16%), and linolenic acids (6%), while the protein content of SB juice varied from 52–766 mg/100 g, with 42–46% of the total essential amino acids. SB juice also contains 2.7–5.3 g of sugar per 100 mL, dominated by glucose, fructose, and xylose [14]. The lowest moisture content of freeze-dried apples was found in FA (6.39 g/100 g), which was comparable to 6.8 g/100 g of freeze-dried Braeburn apple slices [52]. A similar result was also found by Wang et al. [6], who observed a slightly higher moisture content in dried apples treated by OD with different fruit juices, in comparison to that of dried untreated apples. The ash content of 4% CaL was about 7–8 times higher than the controls, which was in accordance with the calcium content of the product (Figure 1A). Although inulin was not involved in the suggested conditions, the 4% CaL sample still possessed significantly higher soluble dietary fiber (SDF), as well as insoluble (IDF) and total dietary fiber (TDF), than the controls (*p* < 0.05), which could be due to the contribution of SB juice. The higher dietary fiber of 4% CaL, especially SDF, might be due to the pectin content in SB, which ranged from 0.21 to 0.68 g/100 g of fresh weight [49]. The 4% CaL treatment also possessed 285.34 kcal/100 g of product, which was about 15.5–17.9% lower than the energy values of FA + AA and FA, respectively. This, then, has the potential to be an alternative functional food or healthy snack with lower calories, reducing the risk of osteoporosis.

## 3. Materials and Methods

### 3.1. Raw Materials and Chemicals

Apples (*Malus domestica* cv. Gala) were acquired in September 2022 from organic farm ‘Dębski Sad’, Rabowice, Poland, and were then transported to the laboratory and processed immediately. The apples were washed, cleaned, cored, peeled (2 mm thick peel), and sliced using an apple peeler and slicer to obtain the C-shaped flesh slices (5 mm thick). The sliced apple flesh was dipped in a solution of 1% ascorbic acid (Stanlab sp. z o.o., Lublin, Poland) for 2 min at a sample–solution ratio of 1:4 to prevent early enzymatic browning, then blotted with absorbing paper and frozen (−40 °C) until further analysis. The prepared flesh had total soluble solids of 10.13 ± 0.06 °Brix (PAL-1 Atago, Fukaya-shi, Japan). The fresh apple flesh (13.27 ± 0.06 °Brix) without dipping in ascorbic acid was also prepared and frozen (−40 °C).

Pasteurized, 100% sea buckthorn (*Hippophae rhamnoides*) juice not from concentrate (7.37 ± 0.06 °Brix) was purchased from Oleofarm sp. z o.o., Wrocław, Poland. Inulin (Orafti HSI) was purchased from Hortimex Plus sp. z o.o., Konin, Poland. Food-grade calcium lactate was purchased from Agnex sp. z o.o., Białystok, Poland.

Chemicals, including 2,2′-Azino-bis(3-ethylbenzothiazoline-6-sulfonic acid) diammonium salt (ABTS), 2,2-Diphenyl-1-picrylhydrazyl (DPPH), 2,2′-Azobis(2-methylpropionamidine) dihydrochloride (AAPH), fluorescein sodium salt, (±)-6-Hydroxy-2,5,7,8-tetramethylchromane-2-carboxylic acid (Trolox), Folin–Ciocalteu’s phenol reagent, gallic acid, 65% nitric acid, methanol, potassium persulfate, sodium carbonate, lanthanum (III) chloride, monopotassium phosphate, disodium phosphate, polyvinylpolypyrrolidone (PVPP), catechol, and guaiacol, were purchased from Sigma-Aldrich, Hamburg, Germany. An ACL reagent kit (lipid-soluble substances) and ACW reagent kit (water-soluble substances) for the photochemiluminescence (PCL) assay were purchased from Analytik Jena, Jena, Germany. The solvents for the ultra-performance liquid chromatography (UPLC) method were bought from Sigma Aldrich, Hamburg, Germany. The standards for the phenolic and carotenoid compounds were obtained from Extrasynthese (Genay, France).

### 3.2. Osmotic Dehydration or Impregnation

The research plan was divided into two parts. In the first part, the effect of various osmotic solution compositions, such as sea buckthorn (SB) juice concentrations (0, 50, and 100% *w*/*w* in water) and inulin–SB juice ratios (0:100, 15:85, 30:70), and process parameters, such as temperature and time (Table 1), on the TPC and antioxidant activities of treated apple flesh was observed. The concentration of calcium lactate (CaL) was controlled at 0% in this first part. For this part, 50 g of frozen apple slices, after dipping in 1% ascorbic acid, were placed in a tightly closed polyethylene terephthalate (PET) jar. The freezing process may rupture the cellular structure of the flesh, which can improve the incorporation of substances. The osmotic solution with the abovementioned composition was added to the PET jar at sample–solution ratio of 1:5. This ratio was chosen to maintain the concentration of solutions during the treatments. The samples were then placed in a water bath (Laboplay SWB 22N, Bytom, Poland) with continuous shaking (150 rpm and a vibration amplitude of 14 mm) at 30 and 50 °C for 30, 60, and 120 min. The treated samples were drained and blotted using absorbing paper and frozen (−40 °C) for 24 h. Afterwards, the samples were freeze dried for 48 h. Freeze-dried fresh apple (FA), freeze-dried fresh apple after dipping in 1% ascorbic acid (FA + AA), as well as freeze-dried treated apples (Table 1) were ground to powder and were ready for analysis. The OD or impregnation process was conducted in triplicate. The optimization of process conditions to obtain the highest TPC and antioxidant activities (ABTS, DPPH, ORAC, PCL) was conducted using Design Expert 13 (Stat-Ease, Minneapolis, MN, USA) (Section 3.4).

In the second part, the treatment process was similar to the first part, only that in this part, the optimized conditions to obtain the highest ORAC value were used (93.8% of SB juice, 0:100 inulin–SB juice ratio, 30 °C, 120 min, Section 2.1.1.), noting that ORAC is more relevant to in vivo conditions because it utilizes peroxyl radical as the biologically relevant free radical source, reflecting the major mechanisms of antioxidant action regarding cell protection [53]. CaL at a concentration of 0, 1, 2, 4, 6% (*w*/*w*) was then added to the osmotic solution. This range of CaL concentration was chosen based on previous studies that showed that it significantly increased the calcium content of treated fruits and affected the mass transfer of water and solutes [4,31]. In this second part, the effect of the addition of CaL on the TPC, antioxidant activities, and calcium content of freeze-dried products was studied.

### 3.3. Analytical Methods

#### 3.3.1. Extraction of Antioxidants

For extraction of antioxidants, the freeze-dried samples in powder form were weighed and extracted in 80% methanol (*w*/*w* in water), at a sample–solvent ratio of 1:20. The samples were shaken in a water bath at 50 °C for 2 h. Afterwards, the samples were cooled down, centrifuged (4 °C, 12,000× *g*, 10 min), and filtered. The extraction was conducted in duplicate. The extracts were used for determination of total phenolic content and antioxidant activities.

#### 3.3.2. Determination of Total Phenolic Content and Antioxidant Activity

Total phenolic content (TPC) was determined following a previous study [54] using a Folin–Ciocalteu reagent. The absorbance of the samples was measured at λ = 725 nm. The results were expressed as mg of gallic acid equivalent (GAE) per 100 g of product.

The antioxidant activity of freeze-dried apple was determined in triplicate using the ABTS, DPPH, ORAC, and PCL methods. The ABTS radical cation scavenging activity was conducted according to a previous study [55]. The degree of reduction in terms of the ABTS radical cation was measured spectrophotometrically at λ = 734 nm after a 6 min incubation of complexes of sample extracts with ABTS and potassium persulfate, expressed as mg of Trolox equivalent (TE) per 100 g of product.

Determination of antioxidant activity using a DPPH assay was determined as described previously [56]. This assay was based on a DPPH solution absorbance decrease at λ = 515 nm in the presence of an antioxidant. The results were expressed as mg of TE/100 g of product.

The oxygen radical absorbance capacity (ORAC) assay was performed according to a previous study [57], which is based on the antioxidant ability to scavenge peroxyl radicals, generated by AAPH using fluorescein solution. The readings were taken at a given excitation (λ = 493 nm) and emission wavelength (λ = 515 nm) on a F-2700 fluorescence spectrophotometer (Hitachi, Tokyo, Japan). The results were presented as mg of TE/100 g of product.

The photochemiluminescence (PCL) assay was conducted using a Photochem^®^ apparatus (Analytik Jena, Jena, Germany), as described previously [58]. This assay is based on the detection of superoxide anion radicals (O_2_^•−^) generated upon exposure to light and the presence of a photosensitizer, chemiluminogenous compound–luminol (5-amino-2,3-dihydro-1,4-phthalazinedione). The antioxidant activities in lipid-soluble (ACL) and water-soluble (ACW) compounds and integral antioxidant capacity (IAC) were presented following the protocol, using the kits provided by the manufacturer. The results were expressed as mg of TE/100 g of product.

#### 3.3.3. Color Measurement

The color of SB juice and the surface of freeze-dried apple samples (from part 1 of the research) before grinding were examined using the CIE L*a*b* scale using a colorimeter (Chroma Meter CR-5, Konica Minolta, Tokyo, Japan). The zero and white calibration was conducted prior to measurement. Results were expressed as L value (lightness), a value (greenness/redness), b value (blueness/yellowness), ΔE (total color difference), BI (browning index), WI (whiteness index), and YI (yellowness index) with the following equations [29]:

(1) $$\Delta E=\sqrt{{\left(\Delta L\right)}^{2}+{\left(\Delta a\right)}^{2}+{\left(\Delta b\right)}^{2}}$$

(2) $$\mathrm{BI}=100\times \frac{x-0.31}{0.172},\;\mathrm{where}\;x=\frac{a+1.75\;L}{5.645L+a-3.012b}$$

(3) $$\mathrm{WI}=100-\sqrt{{\left(100-L\right)}^{2}+a^{2}+b^{2}}$$

(4) $$\mathrm{YI}=142.86\times \frac{b}{L}$$

where for ΔE of freeze-dried samples, the ΔL, Δa, and Δb are the mean difference of L, a, and b values, respectively, between FA + AA and other analyzed samples. The measurements were carried out over six repetitions from each optimized sample in the first part of the research.

#### 3.3.4. Determination of Calcium Content

About 1 g of freeze-dried samples in powder form and SB juice underwent mineralization in a muffle furnace at 450 °C, which was then dissolved in 1 mol/L nitric acid. Each sample was analyzed in triplicate. The samples were diluted in 0.5% LaCl_3_ solution. The calcium content of freeze-dried apples was determined by atomic absorption spectrometry (Hitachi Z2000, Tokyo, Japan) using the air–acetylene flame [59]. The methods were validated by a simultaneous analysis of the reference material (Soya Bean Flour, INCT-SBF-4) with 95.7% accuracy for Ca. The results were expressed as mg of Ca/100 g of product. Deionized water and acid-washed glassware were used in this study.

#### 3.3.5. Analyses of Selected Samples

The selected samples based on the results of the first and second part were further analyzed by ultra-performance liquid chromatography–photodiode array (UPLC-PDA, Waters Corporation, Milford, MA, USA), sensory profiling, activity of enzymes, and proximate analysis.

##### Quantification of Phenolic and Carotenoid Composition of Freeze-Dried Apples by UPLC-PDA

The UPLC-PDA method using ACQUITY Ultra Performance Liquid Chromatography (Waters Corporation, Milford, MA, USA) was performed to quantify the phenolic and carotenoid composition of the samples, with extraction and quantification protocols carried out according to the procedure described previously [34,41]. For the extraction of phenolics, about 0.5 g of selected powdered samples were mixed with 5 mL of 70% methanol (*v*/*v* in water) with 2% ascorbic acid and 1% acetic acid, then sonicated for 15 min (35 kHz; RK514H Bandelin Sonorex, Darmstadt, Germany). The sonication was repeated after storage at 4 °C for 24 h. The samples were centrifuged (14,000× *g*, 4 °C, 10 min; MPW-352R, Warszawa, Poland), and the supernatants were filtered through a hydrophilic polytetrafluoroethylene (PTFE) 0.20 µm membrane (Millex Samplicity Filter, Merck, Darmstadt, Germany) and used for analysis. The separations of individual phenolics were performed at 30 °C using an ACQUITY UPLC BEH C18 column (1.7 µm, 2.1 mm × 100 mm, Waters Corporation, Milford, MA, USA). The sample extracts (5 µL) were injected and the elution was completed in 15 min with a flow rate of 0.420 mL/min. The mobile phase was solvent A (2% formic acid, *v*/*v*) and solvent B (100% acetonitrile). The program started with gradient elution with 98–65% of solvent A (to 12 min) and then 0% (to 14 min); the gradient returned to the initial composition (98% of A) until the 15 min mark, when it was held constant to re-equilibrate the column. The PDA spectra were measured at 280 (flavan-3-ols and dihydrochalcones), 320 (phenolic acids), and 360 nm (flavonols).

The whole extraction procedure for carotenoids was conducted in dark conditions. About 0.4 g of selected powdered samples with the addition of 10% MgCO_3_ was shaken continuously with 4 mL hexane–acetone–methanol (2:1:1, *v*/*v*/*v*) with 1% butylated hydroxytoluene (300 rpm, 15 min; DOS-10L Digital Orbital Shaker, ELMI, Riga, Latvia). The samples were centrifuged (same as with the phenolics extraction), and supernatants were collected. The extraction was repeated two to three times. The combined supernatants were evaporated (XcelVap, Biotage, Uppsala, Sweden) and the residues were diluted in methanol–tetrahydrofuran (4:1, *v*/*v*). Before injection, the samples were filtered through a hydrophilic membrane (as previously described) and used for analysis. The ACQUITY UPLC BEH RP C18 column (1.7 µm, 2.1 mm × 100 mm, Waters Corporation, Milford, MA, USA), protected by a C18 guard column, was operated at 32 °C. The injection volume of sample extracts was 10 µL, and the flow rate was 0.500 mL/min. The mobile phase was solvent A (0.1% formic acid) and solvent B (7:3 acetonitrile–methanol, *v*/*v*). The gradient elution started with 25% A (to 0.60 min); 4.9% A (to 6.50 min); 0% A (to 13.60 min); and 25% A (to 16.60 min). The analysis was monitored at a wavelength of 450 nm.

The retention times and spectra were compared to those of standards. The results of the UPLC-PDA analysis were determined as the average of two replicates and expressed as mg per 100 g of freeze-dried product.

##### Sensory Profiling of Freeze-Dried Apples

The sensory profiling and overall acceptance evaluation were conducted by 20 trained panelists, using a 10 cm linear scale [60]. In the sensory evaluation room, the panelists were asked to assess the selected samples encoded with three-digit numbers. The attributes for sensory profiling were appearance, aroma, taste, and texture, which descriptors were listed in Table S3. The overall acceptance of samples was also analyzed. The study did not require ethical approval. Participants were informed of the study’s purpose and that their contribution was entirely voluntary; therefore, they could stop the analysis at any time and their responses were anonymous. The authors did not ask respondents for sensitive data or personal information. No formal relationship was used to recruit participants to the study. Mean, variance, and standard deviation were calculated for each attribute of all samples and sessions individually.

##### Activity of Enzymes

The activity of enzymes included assay of polyphenol oxidase (PPO) and peroxidase (POD) activities, which are the enzymes responsible for enzymatic browning. For the extraction, 0.3 g of freeze-dried powdered samples was mixed vigorously with 2 mL of 0.067 mol/L of Sorensen’s phosphate buffer solution (SPBS; pH 7.0, determined previously as the optimal pH in freeze-dried apple for both PPO and POD activities) containing 0.02 g of PVPP. The extracts were then shaken for 30 min at 1400 rpm and centrifuged at 12,000× *g*. The supernatants were collected and used for further analysis. The whole extraction process for enzymes was carried out at 4 °C [61].

For the PPO activity, the final reaction mixture contained 50 µL of extract, 900 µL of 0.067 mol/L SPBS (pH 7.0), and 50 µL of 0.5 mol/L catechol as the substrate. For the POD activity, the final reaction mixture contained 100 µL of extract, 800 µL of 0.067 mol/L SPBS (pH 7.0), 50 µL of 0.04 mol/L H_2_O_2_, and 50 µL of 0.04 mol/L guaiacol as the substrate. The changes in the absorbance at 420 and 470 nm (DA) were monitored at 25 °C using a UV-Vis spectrophotometer (UV-1280, Shimadzu, Kyoto, Japan) for PPO and POD activity, respectively. One unit (U) of enzyme activity is defined as the amount of enzyme that changes an absorbance 0.001 per minute under the specified conditions. The PPO and POD activities were expressed in unit per g of product (U/g product).

##### Proximate Analysis of Freeze-Dried Apples

Proximate analysis of selected samples included protein, lipid, carbohydrate, ash, moisture, and fiber. The Kjeldahl method (Kjeltec-2200 System, Foss Tecator, Hoeganaes, Sweden) was applied for protein content determination (PN-75/A-04018:1975) [62]. The Soxhlet method was applied for lipid content determination (PN-EN ISO 3947:2001) [63]. Moisture and ash contents were determined following methods of the Association of Official Agricultural Chemists [64]. The soluble (SDF), insoluble (IDF), and total dietary fiber (TDF) contents were determined according to the Asp method [65]. The carbohydrate content was calculated by subtracting 100 from the sum of protein, lipid, TDF, moisture, and ash content per 100 g of product. The energy value, expressed in kcal/100 g product, was calculated using the conversion factors described in Regulation (EU) No. 1169/2011.

### 3.4. Response Surface Methodology and Statistical Analysis

A combined experimental design (mixture–process) was used to evaluate the effect of three mixture components (sea buckthorn juice, water, and inulin) and two process factors (time and temperature) on the antioxidant properties of the product by employing Design Expert 13 (Stat-Ease, USA). A randomized design was implemented using a Response Surface Cubic model with two-factor interactions (RSpCub × 2FI), totaling 162 experimental runs without blocking. The data for treated samples from Table S1, particularly SB0_I0, SB0_I15, SB0_I30, SB50_I0, SB50_I15, SB50_I30, SB100_I0, SB100_I15, and SB100_I30, treated at different times (30, 60, 120 min) and temperatures (30 and 50 °C) were analyzed (Table S1, Table 1). Each experimental run was replicated in triplicate to ensure data reproducibility and robustness.

Independent variables:

- Mixture components:
  ○ SB juice: 0–250 g;
  ○ Water: 0–250 g;
  ○ Inulin: 0–75 g;
  ○ Total mixture mass fixed at 250 g.
- Process factors:
  ○ Time: 30–120 min;
  ○ Temperature: 30–50 °C.

Dependent variables (responses):

- Total phenolic content (TPC, mg GAE/100 g product).
- Antioxidant activity assays:
  ○ ABTS (mg TE/100 g product);
  ○ DPPH (mg TE/100 g product);
  ○ ORAC (mg TE/100 g product);
  ○ PCL-ACL, PCL-ACW (mg TE/100 g product).

Multiple regression analysis based on response surface methodology (RSM) was applied to develop predictive polynomial regression models describing the relationships between independent variables (mixture components and process factors) and dependent variables (antioxidant responses). Model fitting and validation included calculation of regression coefficients, analysis of variance (ANOVA), and determination of prediction and confidence intervals (95% confidence level) to assess the reliability and accuracy of the developed models. The final models were used to predict optimal combinations of mixture components and processing parameters for maximizing antioxidant activities. *t*-test and one-way ANOVA followed by Tukey’s post hoc test were performed using Statistica 13.1 (StatSoft Inc., Kraków, Poland), noting that a significant difference was considered when *p* < 0.05. The Pearson correlation coefficient was determined between antioxidant activities and TPC of the samples.

## 4. Conclusions

The development of freeze-dried apples impregnated with SB juice, inulin, and CaL at different temperatures and times was studied. Freeze-dried apples prepared by impregnation using 93.8% SB juice, a 0:100 inulin–SB juice ratio, 30 °C, 120 min, with the addition of 4% CaL (also called 4% CaL treatment), possessed a minimum yet acceptable loss of antioxidant properties and increased calcium content (2209.13 mg Ca/100 g). With the assumption of 25% calcium absorption and RDA of 1000 mg of Ca/day for adults, consuming 100 g of freeze-dried apples prepared with 4% CaL could fulfill 55.2% of the RDA. The results from UPLC-PDA indicated that the impregnation process using SB juice and CaL, as well as dipping in ascorbic acid, affected the compositional changes in phenolics and carotenoids of the final product. The 4% CaL treatment also produced a product with lower PPO and POD activities, moderate sensory acceptability with soft texture, and better nutritional values with lower calories when compared to FA and FA + AA. This product has the potential to be an alternative functional food product with respect to reducing the risk of osteoporosis and improving the intake of calcium and antioxidants. Thanks to the beneficial bioactive compounds in the freeze-dried product (4% CaL treatment), it can be beneficial not only for osteoporosis but for general health. In practice, the functional food industry may apply or further improve the suggested conditions to produce the product in a sustainable way, while consumers may include this product in their diet, either as a daily healthy snack or as an alternative food, noting that food diversification is always encouraged to support sustainability.

There are some improvements that could be further studied. Firstly, a lower ratio of inulin–SB juice than 15:85 could be considered not only to potentially minimize the loss of antioxidant activities of the freeze-dried apple, but also to improve the prebiotic effect, calcium absorption, and sensory properties of the end product. Secondly, impregnation using non-conventional technologies, absorption or bioaccessibility of calcium and antioxidants via an in vitro digestion model or in vivo studies, as well as a storage study to observe the stability of bioactive compounds should be conducted. Thirdly, the utilization of potential fruit juices other than SB juice could also be investigated via impregnation or OD processes, noting that every region has its own potential natural resources and thus could also support food diversification in the future.

## Figures and Tables

**Table 1:** Sample preparation for the first part of the research.

Code of Sample	Name of Sample	SB Juice Concentration (*w*/*w* in Water)	Inulin–SB Juice Ratio in Solution (*w*/*w*)	Osmotic Solution Components in Grams During OD (Total Mass = 250 g)			Total Soluble Solid of Osmotic Solution [°Brix]	Time of OD [min]	Temperature of OD [°C]
				SB Juice	Water	Inulin			
FA	Freeze-dried fresh apple			-	-	-	-	-	-
FA + AA	Freeze-dried fresh apple + 1% ascorbic acid			-	-	-	-	-	-
SB0_I0	OD of fresh apple + 1% ascorbic acid (50 g) with freeze drying	0%	0:100	0.00	250.00	0.00	0.00 ± 0.00	30, 60, 120	30, 50
SB0_I15			15:85	0.00	212.50	37.50	13.90 ± 0.00		
SB0_I30			30:70	0.00	175.00	75.00	28.43 ± 0.06		
SB50_I0		50%	0:100	125.00	125.00	0.00	3.83 ± 0.06		
SB50_I15			15:85	106.25	106.25	37.50	17.37 ± 0.06		
SB50_I30			30:70	87.50	87.50	75.00	31.53 ± 0.06		
SB100_I0		100%	0:100	250.00	0.00	0.00	7.37 ± 0.06		
SB100_I15			15:85	212.50	0.00	37.50	20.40 ± 0.10		
SB100_I30			30:70	175.00	0.00	75.00	33.43 ± 0.15		

SB, sea buckthorn; OD, osmotic dehydration.

**Table 2:** Optimized conditions to obtain optimized TPC and antioxidant activities in freeze-dried samples and their validations.

Optimized Conditions *		TPC	ABTS	DPPH	ORAC	PCL-ACL	PCL-ACW
SB juice [g]		170.35	225.31	207.35	234.51	246.92	250.00
Water [g]		79.65	24.69	42.65	15.49	3.08	0.00
Inulin [g]		0.00	0.00	0.00	0.00	0.00	0.00
Time [min]		73.54	119.37	117.72	120.00	120.00	117.82
Temperature [°C]		38.82	30.11	31.33	30.00	30.00	30.40
**Freeze-dried samples**		**TPC [mg GAE/** **100 g product]**	**ABTS [mg TE/** **100 g product]**	**DPPH [mg TE/** **100 g product]**	**ORAC [mg TE/** **100 g product]**	**PCL-ACL [mg TE/** **100 g product]**	**PCL-ACW [mg TE/** **100 g product]**
FA		301.08 ± 13.79 a	449.33 ± 17.37 a	425.99 ± 18.35 b	3423.52 ± 52.93 a	950.62 ± 28.04 b	613.59 ± 5.76 b
FA + AA		623.81 ± 21.06 c	784.18 ± 16.11 b	877.35 ± 30.93 c	3579.68 ± 49.61 b	1138.25 ± 26.18 c	762.84 ± 24.61 c
Optimized conditions **	Validation mean value	427.35 ± 1.59 b	464.54 ± 21.35 a	325.03 ± 0.80 a	3532.58 ± 17.30 b	677.05 ± 6.25 a	471.29 ± 5.11 a
	Predicted mean value	498.39	447.08	324.43	3627.31	668.41	465.28
	95% confidence interval	480.75–516.66	427.23–467.36	311.65–337.45	3356.32–3908.84	644.44–692.38	425.72–506.62
	95% prediction interval	449.35–551.47	413.71–480.35	289.08–361.33	2920.17–4392.88	611.31–725.51	377.01–560.78
Code of samples		Opt_TPC	Opt_ABTS	Opt_DPPH	Opt_ORAC	Opt_PCL-ACL	Opt_PCL-ACW

SB, sea buckthorn. * The total mass of SB juice, water, and inulin in optimized conditions is 250 g. ** Optimized conditions mean that the TPC and antioxidant activities were obtained from freeze-dried samples prepared according to their respective optimized conditions. Different lowercase letters indicate significant differences (*p* < 0.05) between controls (FA and FA + AA) and validation mean values within the same column.

**Table 3:** The appearance and color analysis of freeze-dried apples: FA, FA + AA, and apples treated using optimized TPC and antioxidant activities’ conditions.

Treatment	Appearance	L Value	A Value	b Value	ΔE	BI	WI	YI
FA	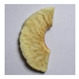	70.96 ± 0.52 d	5.99 ± 0.45 b	29.26 ± 0.63 a	11.17 ± 0.54 a	58.13 ± 1.90 b	58.33 ± 0.57 e	58.92 ± 1.25 b
FA + AA	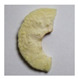	81.06 ± 0.37 e	1.58 ± 0.10 a	27.57 ± 0.27 a	-	41.97 ± 0.53 a	66.51 ± 0.23 f	48.58 ± 0.42 a
Opt_TPC	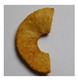	62.19 ± 0.49 c	20.18 ± 0.27 c	50.74 ± 0.13 d	35.20 ± 0.44 b	165.13 ± 2.70 c	33.58 ± 0.41 d	116.57 ± 1.08 c
Opt_ABTS	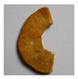	52.27 ± 1.90 b	22.31 ± 0.47 de	48.56 ± 1.44 cd	41.28 ± 0.53 d	208.94 ± 4.42 e	28.31 ± 0.41 b	132.76 ± 1.55 e
Opt_DPPH	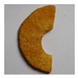	53.74 ± 2.36 b	20.21 ± 0.37 c	47.43 ± 0.57 bc	38.61 ± 1.57 c	190.16 ± 11.25 d	30.71 ± 1.32 c	126.25 ± 4.19 d
Opt_ORAC	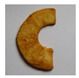	47.74 ± 2.25 a	23.79 ± 1.42 f	48.72 ± 3.09 cd	45.43 ± 0.78 e	248.64 ± 12.57 f	24.61 ± 1.16 a	145.74 ± 4.36 f
Opt_PCL-ACL	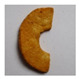	47.64 ± 1.36 a	23.11 ± 0.73 ef	47.38 ± 2.16 bc	44.48 ± 0.43 e	237.00 ± 7.23 f	25.67 ± 0.70 a	142.02 ± 2.50 f
Opt_PCL-ACW	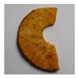	45.14 ± 0.13 a	21.9 ± 0.11 d	45.71 ± 0.11 b	45.08 ± 0.18 e	244.59 ± 2.17 f	25.31 ± 0.17 a	144.69 ± 0.68 f

ΔE is the total color difference between FA + AA and related samples. Different lowercase letters indicate significant differences (*p* < 0.05) between samples within the same column.

**Table 4:** The effect of calcium lactate addition on the TPC and antioxidant activities of freeze-dried apples treated using Opt_ORAC conditions (93.8% SB juice in water, 0:100 inulin–SB juice ratio, 30 °C, 120 min).

Treatment	TPC [mg GAE/100 g Product]	ABTS [mg TE/100 g Product]	DPPH [mg TE/100 g Product]	ORAC [mg TE/100 g Product]	PCL-ACL [mg TE/100 g Product]	PCL-ACW [mg TE/100 g Product]	PCL-IAC [mg TE/100 g Product]
0% CaL	453.33 ± 8.45 d	525.43 ± 24.91 d	346.51 ± 7.94 d	3532.58 ± 17.30 d	710.32 ± 11.84 c	601.40 ± 15.42 d	1311.72 ± 18.98 d
1% CaL	369.68 ± 8.98 c	425.26 ± 3.97 c	307.24 ± 1.90 c	2745.31 ± 177.85 c	583.02 ± 29.29 b	447.85 ± 3.37 c	1030.87 ± 26.24 c
2% CaL	347.41 ± 11.25 bc	394.12 ± 14.29 bc	285.48 ± 1.09 b	2474.84 ± 131.01 bc	503.19 ± 20.62 a	314.31 ± 6.94 b	817.50 ± 21.11 b
4% CaL	331.02 ± 13.99 b	385.69 ± 3.27 b	281.76 ± 7.25 b	2275.52 ± 37.04 b	475.81 ± 8.63 a	282.12 ± 7.46 a	757.93 ± 15.92 ab
6% CaL	280.46 ± 3.79 a	333.18 ± 6.67 a	262.82 ± 2.19 a	1942.63 ± 86.43 a	461.80 ± 25.31 a	265.86 ± 3.01 a	727.66 ± 28.25 a

CaL, calcium lactate. PCL-IAC is the sum of PCL-ACL and PCL-ACW. Different lowercase letters indicate significant differences (*p* < 0.05) between samples within the same column.

**Table 5:** UPLC-PDA quantification data for phenolic and carotenoid composition in freeze-dried apples: FA, FA + AA, and apples treated using Opt_ORAC conditions (93.8% SB juice in water, 0:100 inulin–SB juice ratio, 30 °C, 120 min) with the addition of 4% CaL.

Compounds	No. Compound	Total Content [mg/100 g Product]		
		FA	FA + AA	4% CaL
**PHENOLICS**				
**Phenolic acids**				
p-Coumaric acid-*O*-hexoside	1	30.71 ± 0.09 c	35.3 ± 0.03 b	13.04 ± 0.17 a
Ferulic acid-*O*-hexoside	2	3.88 ± 0.08 b	3.91 ± 0.05 b	0.92 ± 0.10 a
**Sum of phenolic acids**		34.59 ± 0.17 b	39.21 ± 0.02 c	13.96 ± 0.08 a
				
**Flavan-3-ols**				
(+)-Catechin	3	3.60 ± 0.18 a	6.25 ± 0.13 b	17.97 ± 0.19 c
Procyanidin B1	4	nd	nd	17.88 ± 0.04
(-)-Epicatechin	5	10.59 ± 0.63 b	13.06 ± 0.01 c	3.03 ± 0.10 a
Procyanidin C1	6	32.04 ± 1.32 b	37.21 ± 0.57 c	5.48 ± 0.33 a
**Sum of flavan-3-ols**		46.24 ± 0.51 a	56.53 ± 0.43 b	44.36 ± 0.58 a
				
**Dihydrochalcones**				
Phloretin-2-*O*-xyloglucoside	7	2.15 ± 0.00 c	1.61 ± 0.02 a	1.81 ± 0.04 b
Derivative of dihydrochalcones	8	3.83 ± 0.08 b	3.48 ± 0.11 a	nd
Phloretin-2-*O*-glucoside	9	1.47 ± 0.31 a	1.36 ± 0.07 a	nd
**Sum of dihydrochalcones**		7.46 ± 0.4 b	6.44 ± 0.2 b	1.81 ± 0.04 a
				
**Flavonols**				
Isorhamnetin-3,7-*O*-dihexoside	10	nd	nd	4.28 ± 0.08
Isorhamnetin-3-*O*-sophoroside-7-*O*-rhamnoside	11	nd	nd	1.93 ± 0.05
Quercetin-3-*O*-(dirhamnosyl)hexoside	12	nd	nd	3.68 ± 0.20
Quercetin-3-galactoside-7-*O*-rhamnoside	13	nd	nd	1.02 ± 0.20
Quercetin-3-*O*-rutinoside	14	nd	nd	1.24 ± 0.06
Isorhamnetin-3-*O*-glucoside-7-*O*-rhamnoside	15	nd	nd	2.65 ± 0.17
Quercetin-3-*O*-glucoside	16	nd	nd	4.56 ± 0.09
Isorhamnetin-3-*O*-(2-rhamnosyl)hexoside	17	nd	nd	2.50 ± 0.00
Kaempferol-3-*O*-hexoside-7-*O*-rhamnoside	18	nd	nd	4.06 ± 0.23
Isorhamnetin-3-*O*-(rhamnosyl)hexoside	19	nd	nd	0.72 ± 0.00
Isorhamnetin-3-*O*-rutinoside	20	nd	nd	8.23 ± 0.00
Quercetin-3-*O*-rhamnoside	21	1.07 ± 0.08 a	0.96 ± 0.04 a	1.81 ± 0.03 b
Isorhamnetin-3-*O*-glucoside	22	nd	nd	8.72 ± 0.02
Kaempferol-3-*O*-rutinoside	23	nd	nd	0.63 ± 0.02
Isorhamnetin-3-*O*-rhamnoside	24	nd	nd	0.32 ± 0.05
**Sum of flavonols**		1.07 ± 0.08a	0.96 ± 0.04a	46.36 ± 0.39 b
				
**Total phenolics**		89.36 ± 0.36 a	103.14 ± 0.26 b	106.49 ± 0.86 c
**CAROTENOIDS**				
Lutein isomer	25	0.28 ± 0.01 a	0.25 ± 0.04 a	nd
All-trans-lutein	26	0.19 ± 0.12 a	0.17 ± 0.02 a	nd
Zeaxanthin isomer	27	nd	nd	9.75 ± 1.71
Sum of carotene	28	1.75 ± 0.10 a	1.76 ± 0.05 a	28.57 ± 0.73 b
Phytofluene	29	0.07 ± 0.00 a	0.05 ± 0.01 a	0.31 ± 0.00 b
**Total carotenoids**		2.29 ± 0.24 a	2.23 ± 0.02 a	38.63 ± 2.45 b

nd, not detected. Different lowercase letters indicate significant differences (*p* < 0.05) between samples within the same row.

**Table 6:** Activity of enzymes and proximate analysis of freeze-dried apples: FA, FA + AA, and apples treated using Opt_ORAC conditions (93.8% SB juice in water, 0:100 inulin–SB juice ratio, 30 °C, 120 min) with the addition of 4% CaL.

Analyses	Unit	FA	FA + AA	4% CaL
**Activity of enzymes**				
PPO activity	U/g product	3894.81 ± 247.07 c	3358.52 ± 175.90 b	432.59 ± 28.57 a
POD activity	U/g product	717.78 ± 26.94 b	1642.22 ± 36.72 c	407.41 ± 12.83 a
**Proximate analysis**				
Protein	g/100 g product	4.38 ± 0.08 b	3.52 ± 0.19 a	5.14 ± 0.38 c
Lipid	g/100 g product	0.53 ± 0.03 a	0.68 ± 0.03 a	3.64 ± 0.18 b
Carbohydrate	g/100 g product	75.20 ± 0.27 b	73.29 ± 0.36 b	48.96 ± 1.47 a
Ash	g/100 g product	1.27 ± 0.11 a	1.12 ± 0.05 a	8.81 ± 0.29 b
Moisture	g/100 g product	6.39 ± 0.29 a	8.95 ± 0.34 b	15.36 ± 0.77 c
TDF	g/100 g product	12.22 ± 0.29 a	12.44 ± 0.62 a	18.09 ± 0.31 b
SDF	g/100 g product	2.57 ± 0.62 a	3.05 ± 0.47 a	4.18 ± 0.06 b
IDF	g/100 g product	9.65 ± 0.48 a	9.39 ± 0.36 a	13.90 ± 0.37 b
Energy value	kcal/100 g product	347.53	338.24	285.34

Carbohydrate was calculated using the formula of 100 g—(mass in g [protein + lipid + ash + moisture + TDF]) in 100 g of product. Energy value was calculated using the conversion based on Regulation (EU) No 1169/2011: carbohydrates—4 kcal/g, protein—4 kcal/g, lipid—9 kcal/g, and fiber (TDF)—2 kcal/g). Different lowercase letters indicate significant differences (*p* < 0.05) between samples within the same row.

## Data Availability

The data that support the findings of this study are available from the corresponding author upon reasonable request.

## Supplementary Materials

The following supporting information can be downloaded at https://www.mdpi.com/article/10.3390/molecules30071504/s1: Table S1: TPC and antioxidant activities of freeze-dried apples, osmotically dehydrated with different concentrations of SB juice (0, 50, 100% in water), inulin–SB juice ratios (0:100, 15:85, 30:70), times (30, 60, 120 min), and temperatures (30, 50 °C); Table S2: Pearson’s correlation coefficients for TPC and antioxidant activities of freeze-dried apples; Table S3: Sensory profiling attributes and descriptors.
