# Supplementary material for: Product Development Study of Freeze-Dried Apples Enriched with Sea Buckthorn Juice and Calcium Lactate
Source: Molecules. 2025 Mar 28;30(7):1504. doi: 10.3390/molecules30071504 (PMC11990202; doi:10.3390/molecules30071504)
Supplement: Supplementary file 1 [file molecules-30-01504-s001.zip › molecules-3539079-supplementary.pdf]

Supplementary Materials

Product Development Study of Freeze-Dried Apples Enriched with Sea Buckthorn Juice and Calcium Lactate

Marcellus Arnold, Wojciech Białas, Bartosz Kulczyński, Ribi Ramadanti Multisona, Joanna Suliburska, Michał Świeca, Aneta Wojdyło, Anna Gramza-Michałowska

Table S1. TPC and antioxidant activities of freeze-dried apples, osmotically dehydrated with different concentrations of SB juice (0, 50, 100% in water), inulin:SB juice ratios (0:100, 15:85, 30:70), times (30, 60, 120 min), and temperatures (30, 50°C).

**30 min, 30°C**							
**Code of sample**	**TPC [mg GAE/ 100 g product]**	**ABTS [mg TE/ 100 g product]**	**DPPH [mg TE/ 100 g product]**	**ORAC [mg TE/ 100 g product]**	**PCL-ACL [mg TE/ 100 g product]**	**PCL-ACW [mg TE/ 100 g product]**	**PCL-IAC [mg TE/ 100 g product]**
FA	301.08 ± 13.79d	449.33 ± 17.37f	425.99 ± 18.35f	3423.52 ± 52.93g	950.62 ± 28.04f	613.59 ± 5.76g	1564.22 ± 25.34f
FA+AA	623.81 ± 21.06h	784.18 ± 16.11g	877.35 ± 30.93g	3579.68 ± 49.61g	1138.25 ± 26.18g	762.84 ± 24.61h	1901.08 ± 48.58g
SB0_I0	187.02 ± 2.53a	175.86 ± 1.67b	182.04 ± 0.57b	1274.53 ± 20.92b	179.11 ± 2.69a	158.13 ± 5.25bc	337.23 ± 7.79b
SB0_I15	180.81 ± 1.88a	134.62 ± 0.04a	160.75 ± 1.45ab	862.93 ± 36.03a	178.66 ± 4.50a	171.08 ± 0.81c	349.74 ± 4.72b
SB0_I30	159.69 ± 4.13a	108.81 ± 3.85a	148.03 ± 4.03a	780.98 ± 32.18a	141.52 ± 1.96a	81.55 ± 1.27a	223.08 ± 1.38a
SB50_I0	382.97 ± 1.41f	312.16 ± 1.97d	255.98 ± 2.58cd	2304.12 ± 101.35e	523.29 ± 2.50e	308.12 ± 8.33e	831.40 ± 6.09e
SB50_I15	267.98 ± 9.43c	232.77 ± 11.84c	250.43 ± 4.44c	1384.10 ± 51.79bc	400.41 ± 15.09d	183.54 ± 5.81c	583.95 ± 20.89d
SB50_I30	218.24 ± 6.34b	172.82 ± 1.44b	186.51 ± 3.46b	1373.09 ± 105.21bc	279.13 ± 6.60b	139.94 ± 4.57b	414.67 ± 7.42c
SB100_I0	447.88 ± 2.08g	374.10 ± 12.53e	289.78 ± 3.50e	2802.31 ± 70.6f	530.39 ± 1.60e	350.69 ± 2.50f	881.09 ± 3.85e
SB100_I15	342.44 ± 9.22e	340.41 ± 2.23d	287.72 ± 1.27de	1921.60 ± 75.7d	525.48 ± 7.02e	311.91 ± 8.33e	837.39 ± 13.35e
SB100_I30	278.36 ± 11.04cd	245.05 ± 14.71c	230.74 ± 5.91c	1508.86 ± 55.86c	328.40 ± 2.83c	214.92 ± 4.89d	543.32 ± 2.08d
**30 min, 50°C**							
**Code of sample**	**TPC [mg GAE/ 100 g product]**	**ABTS [mg TE/ 100 g product]**	**DPPH [mg TE/ 100 g product]**	**ORAC [mg TE/ 100 g product]**	**PCL-ACL [mg TE/ 100 g product]**	**PCL-ACW [mg TE/ 100 g product]**	**PCL-IAC [mg TE/ 100 g product]**
FA	301.08 ± 13.79f	449.33 ± 17.37g	425.99 ± 18.35e	3423.52 ± 52.93h	950.62 ± 28.04h	613.59 ± 5.76g	1564.22 ± 25.34h
FA+AA	623.81 ± 21.06h	784.18 ± 16.11h	877.35 ± 30.93f	3579.68 ± 49.61i	1138.25 ± 26.18i	762.84 ± 24.61h	1901.08 ± 48.58i
SB0_I0	159.76 ± 2.38bc	121.20 ± 2.89b	127.27 ± 0.43a	845.46 ± 62.41c	196.86 ± 6.18b	157.79 ± 4.64c	354.65 ± 9.96c
SB0_I15	138.08 ± 4.23ab	99.16 ± 3.43ab	132.49 ± 15.33a	587.66 ± 52.56b	179.04 ± 2.76ab	102.56 ± 3.55b	281.60 ± 1.07b
SB0_I30	119.35 ± 3.00a	77.67 ± 2.22a	122.44 ± 1.53a	413.74 ± 17.99a	143.92 ± 2.05a	62.86 ± 1.19a	206.78 ± 2.64a
SB50_I0	341.02 ± 6.76g	287.48 ± 4.58e	255.71 ± 2.02d	2020.29 ± 111.32g	566.8 ± 23.8fg	289.57 ± 5.41e	856.36 ± 29.13g
SB50_I15	214.65 ± 5.39d	210.77 ± 2.47d	207.46 ± 2.76c	1311.61 ± 46.62e	487.49 ± 7.14e	173.89 ± 11.27c	661.39 ± 17.24e
SB50_I30	174.67 ± 2.06c	152.83 ± 6.68c	167.62 ± 1.28b	890.59 ± 30.23c	297.38 ± 21.18c	118.79.36 ± 0.66b	416.16 ± 20.87c
SB100_I0	356.36 ± 4.25g	327.9 ± 7.45f	277.02 ± 3.82d	2045.50 ± 19.53g	586.03 ± 13.67g	327.08 ± 3.48f	913.11 ± 10.28g
SB100_I15	260.29 ± 0.85e	275.71 ± 4.88e	251.45 ± 3.03d	1604.12 ± 53.76f	530.03 ± 7.47ef	225.20 ± 7.26d	755.23 ± 11.73f
SB100_I30	214.85 ± 0.60d	214.3 ± 7.97d	214.48 ± 4.65c	1151.78 ± 17.45d	354.62 ± 9.09d	167.12 ± 3.51c	521.74 ± 6.89d

Table S1 (continued)

**60 min, 30°C**							
**Code of sample**	**TPC [mg GAE/ 100 g product]**	**ABTS [mg TE/ 100 g product]**	**DPPH [mg TE/ 100 g product]**	**ORAC [mg TE/ 100 g product]**	**PCL-ACL [mg TE/ 100 g product]**	**PCL-ACW [mg TE/ 100 g product]**	**PCL-IAC [mg TE/ 100 g product]**
FA	301.08 ± 13.79f	449.33 ± 17.37f	425.99 ± 18.35g	3423.52 ± 52.93g	950.62 ± 28.04g	613.59 ± 5.76g	1564.22 ± 25.34g
FA+AA	623.81 ± 21.06i	784.18 ± 16.11g	877.35 ± 30.93h	3579.68 ± 49.61g	1138.25 ± 26.18h	762.84 ± 24.61h	1901.08 ± 48.58h
SB0_I0	155.59 ± 3.90b	186.01 ± 7.74b	181.14 ± 2.47bc	560.37 ± 10.86a	133.39 ± 1.17a	122.28 ± 5.51b	255.66 ± 4.59b
SB0_I15	131.37 ± 1.18ab	85.15 ± 0.82a	127.89 ± 0.72a	434.49 ± 17.36a	118.23 ± 1.93a	57.97 ± 2.65a	176.19 ± 3.02a
SB0_I30	117.36 ± 1.43a	73.86 ± 2.52a	120.55 ± 4.58a	397.45 ± 19.25a	109.77 ± 1.96a	57.92 ± 1.04a	167.69 ± 2.93a
SB50_I0	439.80 ± 11.35g	406.31 ± 6.27e	290.07 ± 5.20ef	2229.09 ± 96.32e	537.50 ± 3.26e	418.57 ± 18.34f	956.07 ± 15.09f
SB50_I15	261.07 ± 2.93e	241.14 ± 3.82c	223.30 ± 5.01d	1177.46 ± 25.71c	455.52 ± 17.93d	288.08 ± 1.70e	743.60 ± 19.46e
SB50_I30	183.64 ± 3.11c	182.82 ± 6.32b	176.76 ± 5.15b	781.19 ± 18.29b	270.41 ± 0.69b	120.36 ± 3.60b	390.77 ± 3.53c
SB100_I0	475.67 ± 4.61h	413.59 ± 18.09e	317.51 ± 0.80f	2495.79 ± 139.21f	581.52 ± 12.75f	418.70 ± 30.93f	1000.22 ± 35.49f
SB100_I15	304.92 ± 2.44f	297.81 ± 19.99d	267.64 ± 9.00e	2039.44 ± 128.82e	547.59 ± 8.08ef	239.94 ± 8.47d	787.53 ± 8.73e
SB100_I30	222.11 ± 4.60d	205.69 ± 1.68b	211.66 ± 2.90cd	1393.10 ± 7.32d	344.11 ± 6.35c	169.19 ± 3.97c	513.30 ± 9.93d

**60 min, 50°C**							
**Code of sample**	**TPC [mg GAE/ 100 g product]**	**ABTS [mg TE/ 100 g product]**	**DPPH [mg TE/ 100 g product]**	**ORAC [mg TE/ 100 g product]**	**PCL-ACL [mg TE/ 100 g product]**	**PCL-ACW [mg TE/ 100 g product]**	**PCL-IAC [mg TE/ 100 g product]**
FA	301.08 ± 13.79e	449.33 ± 17.37f	425.99 ± 18.35f	3423.52 ± 52.93h	950.62 ± 28.04f	613.59 ± 5.76g	1564.22 ± 25.34f
FA+AA	623.81 ± 21.06g	784.18 ± 16.11g	877.35 ± 30.93g	3579.68 ± 49.61h	1138.25 ± 26.18g	762.84 ± 24.61h	1901.08 ± 48.58g
SB0_I0	169.69 ± 5.36b	115.47 ± 4.15b	121.49 ± 3.41bc	554.84 ± 23.75b	155.43 ± 0.35b	167.63 ± 4.81d	323.06 ± 4.94b
SB0_I15	120.34 ± 4.50a	56.64 ± 2.85a	94.93 ± 1.95ab	428.84 ± 2.04ab	115.18 ± 1.47a	55.02 ± 0.93a	170.21 ± 0.78a
SB0_I30	102.39 ± 1.99a	46.33 ± 0.84a	82.65 ± 3.98a	282.45 ± 23.74a	107.27 ± 2.32a	38.75 ± 2.59a	146.02 ± 2.82a
SB50_I0	425.59 ± 2.90f	390.96 ± 14.84e	289.85 ± 3.82e	2226.50 ± 47.76f	520.10 ± 1.75e	395.70 ± 4.74f	915.79 ± 5.18e
SB50_I15	226.97 ± 6.75c	206.87 ± 7.49cd	209.09 ± 6.41d	1092.01 ± 78.45d	427.13 ± 18.30d	201.61 ± 11.29e	628.74 ± 25.07d
SB50_I30	172.08 ± 4.03b	133.94 ± 4.70b	149.76 ± 4.94c	772.98 ± 45.33c	258.10 ± 0.69c	107.15 ± 3.09b	365.25 ± 3.78bc
SB100_I0	432.32 ± 4.25f	391.27 ± 12.27e	304.75 ± 2.78e	2410.10 ± 108.32g	531.06 ± 5.25e	370.22 ± 2.41f	901.29 ± 6.30e
SB100_I15	258.10 ± 4.67d	228.66 ± 3.70d	224.99 ± 1.57d	1371.36 ± 18.38e	444.62 ± 6.19d	182.23 ± 5.19de	635.50 ± 12.28d
SB100_I30	208.57 ± 5.95c	184.23 ± 5.27c	196.60 ± 4.08d	1064.80 ± 91.96d	272.03 ± 1.81c	138.64 ± 3.35c	410.67 ± 5.03c

Table S1 (continued)

120 min, 30°C							
**Code of sample**	**TPC [mg GAE/ 100 g product]**	**ABTS [mg TE/ 100 g product]**	**DPPH [mg TE/ 100 g product]**	**ORAC [mg TE/ 100 g product]**	**PCL-ACL [mg TE/ 100 g product]**	**PCL-ACW [mg TE/ 100 g product]**	**PCL-IAC [mg TE/ 100 g product]**
FA	301.08 ± 13.79e	449.33 ± 17.37f	425.99 ± 18.35g	3423.52 ± 52.93g	950.62 ± 28.04g	613.59 ± 5.76h	1564.22 ± 25.34h
FA+AA	623.81 ± 21.06h	784.18 ± 16.11g	877.35 ± 30.93h	3579.68 ± 49.61g	1138.25 ± 26.18h	762.84 ± 24.61i	1901.08 ± 48.58i
SB0_I0	172.13 ± 3.61bc	120.24 ± 3.52a	134.39 ± 1.27a	844.79 ± 28.70a	158.30 ± 3.72b	130.28 ± 7.45b	288.58 ± 7.89b
SB0_I15	145.64 ± 7.60ab	118.43 ± 5.73a	133.02 ± 0.36a	840.15 ± 17.21a	129.53 ± 2.29ab	84.06 ± 2.20a	213.59 ± 3.72a
SB0_I30	126.09 ± 6.09a	91.27 ± 8.30a	119.05 ± 6.03a	644.84 ± 62.31a	112.80 ± 10.21a	69.09 ± 1.42a	181.89 ± 11.16a
SB50_I0	388.42 ± 0.86f	355.76 ± 8.43e	276.43 ± 2.5de	2944.97 ± 22.72f	529.11 ± 7.15e	367.71 ± 9.63f	896.82 ± 16.73f
SB50_I15	280.76 ± 13.71e	237.00 ± 9.26c	251.83 ± 11.82cd	2147.31 ± 204.88d	446.98 ± 17.4d	201.07 ± 4.39d	648.05 ± 17.20d
SB50_I30	199.83 ± 9.10cd	168.41 ± 15.54b	195.43 ± 9.52b	1508.22 ± 52.21b	292.98 ± 2.33c	171.95 ± 1.16c	464.93 ± 3.48c
SB100_I0	471.61 ± 23.53g	427.62 ± 16.97f	315.10 ± 7.82f	3662.73 ± 123.58g	673.13 ± 15.64f	458.37 ± 4.87g	1131.50 ± 20.43g
SB100_I15	310.82 ± 14.51e	279.23 ± 11.61d	291.07 ± 10.23ef	2677.72 ± 130.26e	506.52 ± 3.3e	243.07 ± 10.64e	749.59 ± 8.05e
SB100_I30	226.74 ± 1.48d	196.33 ± 14.5b	221.43 ± 3.73bc	1859.53 ± 42.07c	319.40 ± 5.71c	174.47 ± 3.71cd	493.87 ± 6.30c
**120 min, 50°C**							
**Code of sample**	**TPC [mg GAE/ 100 g product]**	**ABTS [mg TE/ 100 g product]**	**DPPH [mg TE/ 100 g product]**	**ORAC [mg TE/ 100 g product]**	**PCL-ACL [mg TE/ 100 g product]**	**PCL-ACW [mg TE/ 100 g product]**	**PCL-IAC [mg TE/ 100 g product]**
FA	301.08 ± 13.79e	449.33 ± 17.37g	425.99 ± 18.35f	3423.52 ± 52.93i	950.62 ± 28.04f	613.59 ± 5.76h	1564.22 ± 25.34g
FA+AA	623.81 ± 21.06h	784.18 ± 16.11h	877.35 ± 30.93g	3579.68 ± 49.61j	1138.25 ± 26.18g	762.84 ± 24.61i	1901.08 ± 48.58h
SB0_I0	148.91 ± 6.02b	93.52 ± 0.61b	100.03 ± 0.78a	715.05 ± 46.75c	135.93 ± 11.79a	125.42 ± 3.86cd	261.35 ± 15.59b
SB0_I15	129.66 ± 3.70ab	80.41 ± 0.77ab	115.26 ± 1.19a	506.43 ± 13.74b	133.92 ± 1.10a	70.54 ± 0.37b	204.71 ± 1.15ab
SB0_I30	115.80 ± 5.34a	59.42 ± 0.82a	102.33 ± 1.13a	326.49 ± 7.95a	122.26 ± 7.85a	42.93 ± 0.43a	165.19 ± 7.74a
SB50_I0	383.42 ± 13.92f	330.91 ± 13.32e	251.99 ± 5.44d	2224.95 ± 120.60g	510.73 ± 10.35d	283.54 ± 1.98f	793.61 ± 8.88e
SB50_I15	262.46 ± 1.03d	214.71 ± 1.44d	199.12 ± 2.53c	1129.12 ± 82.38e	446.33 ± 25.88c	167.95 ± 5.05e	614.28 ± 29.63d
SB50_I30	184.60 ± 9.56c	146.19 ± 1.59c	156.17 ± 0.53b	718.66 ± 34.74c	271.06 ± 5.47b	104.03 ± 2.02c	375.09 ± 4.95c
SB100_I0	462.77 ± 10.01g	405.11 ± 0.72f	309.65 ± 1.15e	2637.80 ± 17.65h	560.37 ± 5.18e	355.63 ± 2.35g	916.00 ± 7.44f
SB100_I15	283.36 ± 4.66d	233.81 ± 5.18d	216.39 ± 2.76c	1328.69 ± 21.39f	475.88 ± 17.57cd	177.45 ± 1.51e	653.33 ± 17.64d
SB100_I30	209.42 ± 2.57c	167.19 ± 4.49c	166.39 ± 1.91b	904.09 ± 7.98d	285.51 ± 12.66b	128.46 ± 1.19d	413.97 ± 11.63c

PCL-IAC is the sum of PCL-ACL and PCL-ACW. Different lowercases indicate significant difference ($p<0.05$) between controls and treated samples in one column at the same time and temperature.

Table S2. Pearson's correlation coefficient between TPC and antioxidant activities of freeze-dried apples.

	TPC	ABTS	DPPH	ORAC	PCL-ACL	PCL-ACW	PCL-IAC
**TPC**	1.00						
**ABTS**	0.98*	1.00					
**DPPH**	0.94*	0.97*	1.00				
**ORAC**	0.93*	0.92*	0.92*	1.00			
**PCL-ACL**	0.91*	0.93*	0.94*	0.88*	1.00		
**PCL-ACW**	0.96*	0.96*	0.91*	0.91*	0.88*	1.00	
**PCL-IAC**	0.96*	0.97*	0.95*	0.92*	0.98*	0.96*	1.00

Significant correlation ($p<0.05$) is marked by asterisk symbol (*).

Table S3. Sensory profiling attributes and descriptors

Attributes	Descriptors	Line scale	
		0 cm	10 cm
Appearance	Yellowness	light yellow	dark yellow
	Brownness	light brown	dark brown
	Color uniformity	not uniform	uniform
Aroma	Fruity	apple	sea buckthorn
	Grassy	absent	very intense
	Foreign	absent	very intense
Taste	Astringency	absent	very intense
	Sourness	absent	very intense
	Sweetness	absent	very intense
	Bitterness	absent	very intense
	Saltiness	absent	very intense
	Umami	absent	very intense
Texture	Crispiness	absent	very intense
	Hardness	soft	hard
	Adhesiveness to palate	absent	very intense
Overall acceptance		extremely unacceptable	extremely acceptable
